# Management of a Patient With an Anomalous Right Coronary Artery: A Case Report

**DOI:** 10.7759/cureus.73516

**Published:** 2024-11-12

**Authors:** Catherine Raymond, Raquel Rudy, Chris Jacob, Joan Crawford

**Affiliations:** 1 Internal Medicine, Ascension Macomb-Oakland Hospital, Warren, USA; 2 Cardiology, Ascension Macomb-Oakland Hospital, Warren, USA

**Keywords:** anomalous rca, cardiovascular disease, congenital heart abnormality, coronary computed tomoangiography, stable angina pectoris

## Abstract

Coronary artery anomalies are rare congenital defects that involve abnormalities in the origin, course, or termination of the three main epicardial coronary arteries. Due to the variety of aberrant coronary artery defects, the clinical presentation can differ. Anomalous origins of the right coronary artery include the pulmonary trunk, ascending aorta, left sinus of Valsalva, and a course that traverses between the great vessels. Diagnosis is made using multidetector computed tomography coronary angiography or coronary computed tomography angiography. Management may include lifestyle modifications, medications, or invasive surgical interventions. Here, we present the case of a 65-year-old woman with recurrent angina, leading to multiple emergency visits and hospitalizations. The patient was ultimately diagnosed with an anomalous right coronary artery, with a high takeoff originating anteriorly above the sinus of Valsalva and traversing normally after a short segment between the aorta and the main pulmonary artery. This case highlights the complex and controversial management of anomalous coronary arteries and underscores the need for further research to establish optimal, guideline-directed treatment strategies.

## Introduction

Anomalies of the coronary artery are rare congenital defects that involve abnormalities in the origin, course, or termination of the three main epicardial coronary arteries, such as the left anterior descending, left circumflex, or right coronary artery (RCA), which occur in 0.3%-1.3% of the population [1-6]. Anomalous origins of the RCA include the pulmonary trunk, ascending aorta, left sinus of Valsalva, and a course that traverses between the great vessels [2]. Due to the variety of aberrant coronary artery defects, the presentation can range from asymptomatic to sudden cardiac arrest (SCA). Patients may present with a variety of complaints, including chest pain, shortness of breath, dyspnea on exertion, and syncope. Research has shown that risk factors associated with SCA include younger age (typically under 40 years old), exercise-induced ischemia, absence of prodromal symptoms, and an acute takeoff angle from the aorta [4,6]. Management varies from conservative measures, such as pharmaceutical and lifestyle modifications, to surgical interventions, including stents, grafts, or reimplantation [3,4]. We present the case of an older woman with recurrent symptoms of anomalous RCA, who was treated conservatively, highlighting the ongoing controversy regarding the management of anomalous coronary arteries.

## Case presentation

A 65-year-old woman with an underlying anomalous RCA, a history of recurrent episodic stable angina, Hashimoto’s thyroiditis, and hyperlipidemia presented to the emergency department with complaints of intermittent central chest pressure and burning, radiating to her back. The symptoms had started three days prior to presentation, worsened when lying flat at night, and resolved spontaneously without intervention.

**Figure 1:**
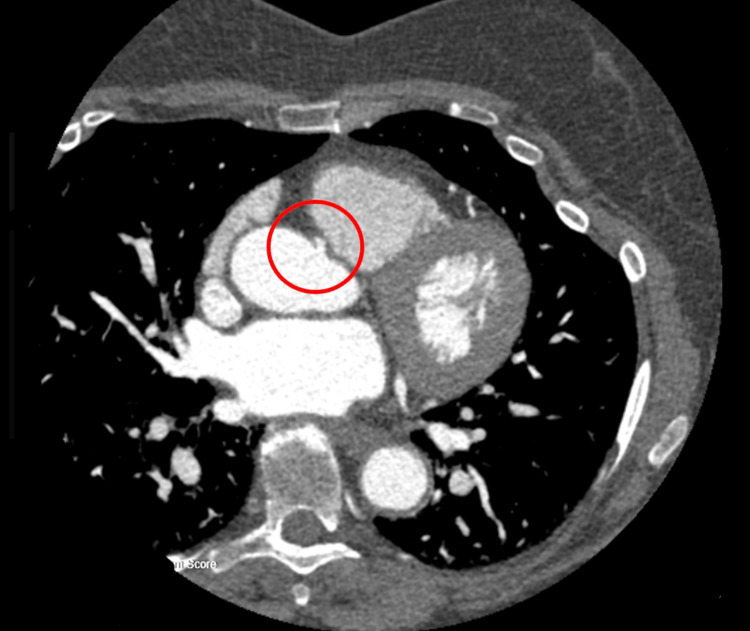
Computed tomography angiography 2D image showing the origin of the anomalous right coronary artery (within the red circle).

**Figure 2:**
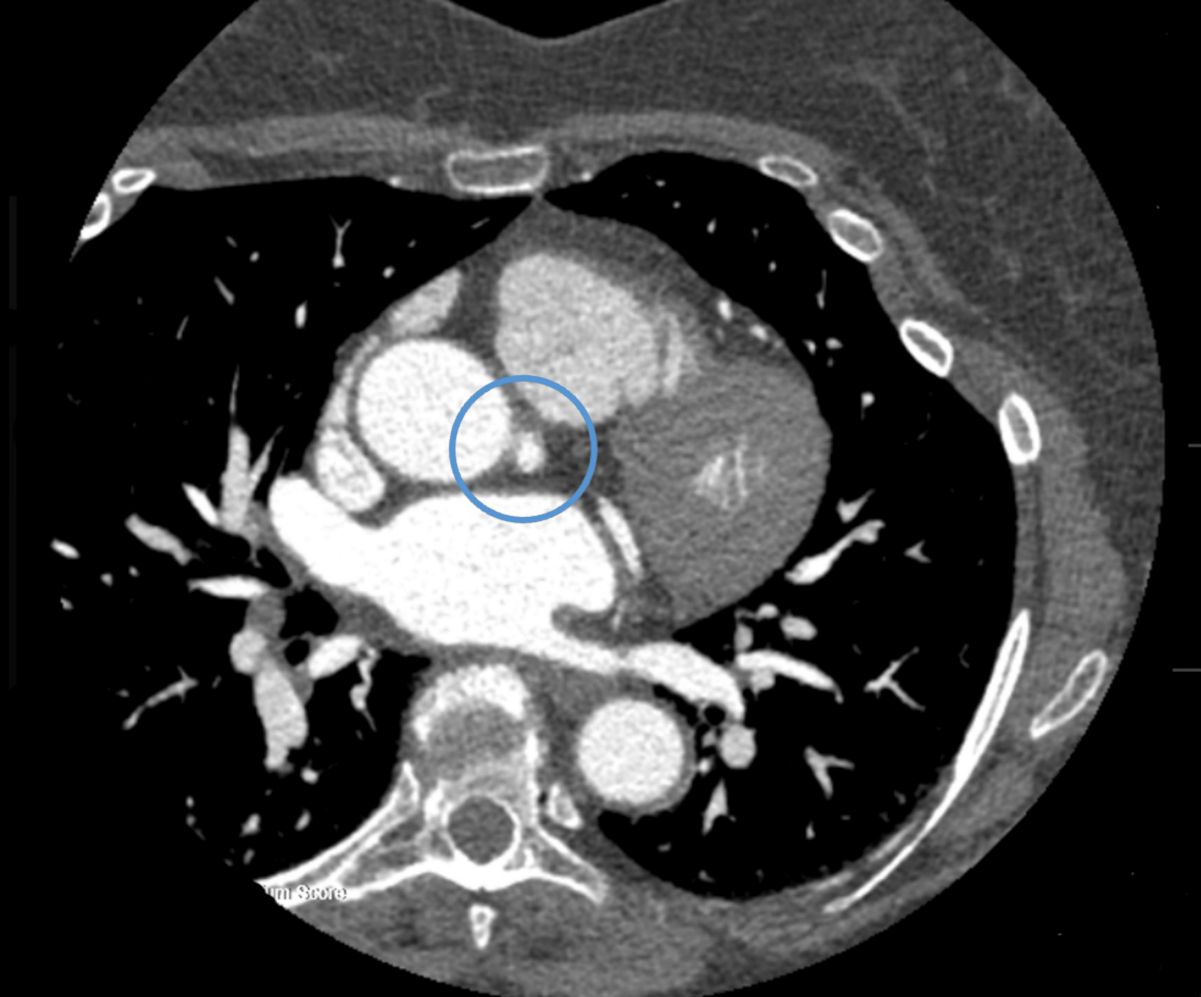
Computed tomography angiography 2D image showing the origin of the left main artery (within the blue circle).

**Figure 3:**
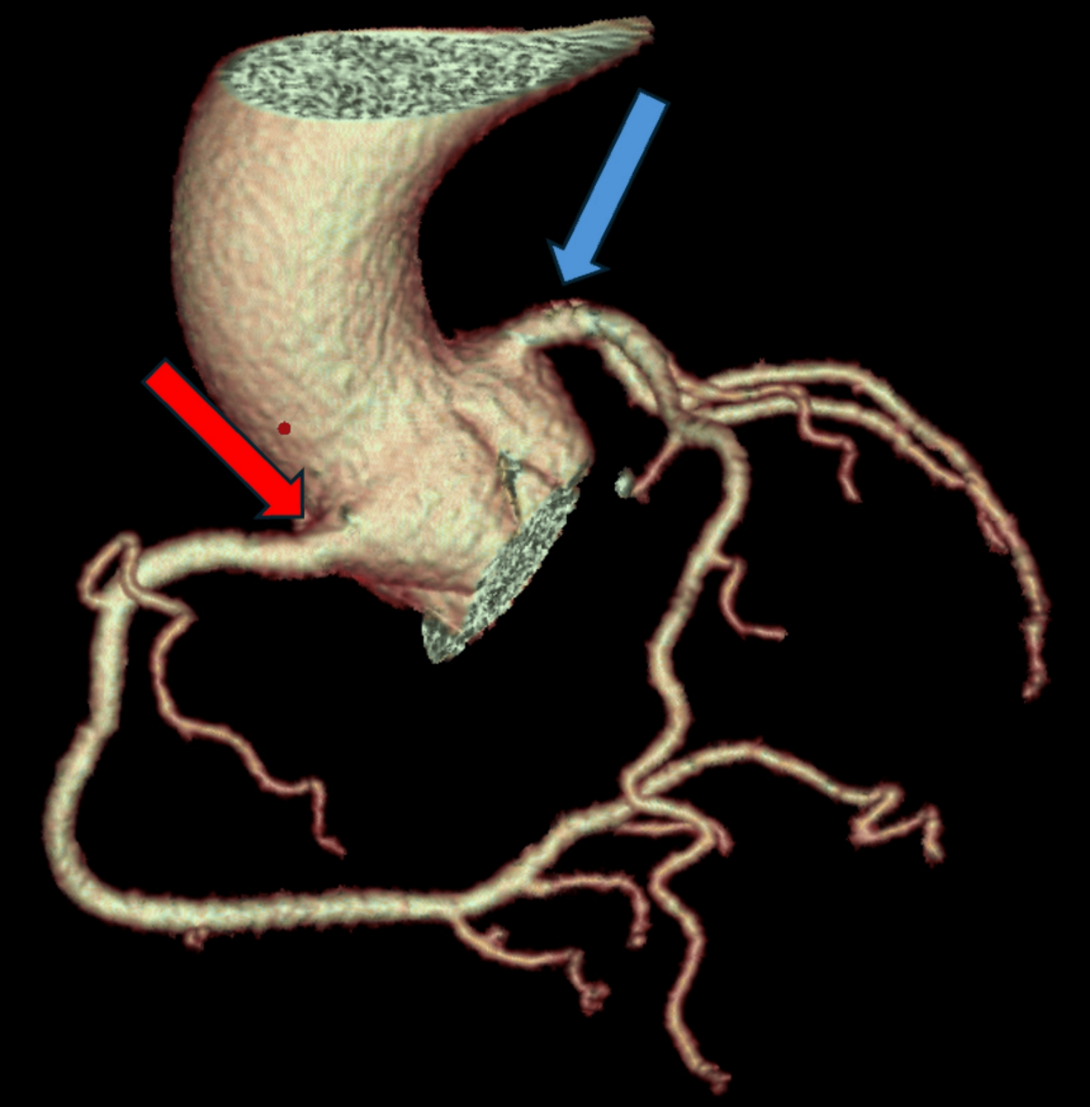
Computed tomography angiography with a 3D-rendered image demonstrating the normal course of the coronary arteries. The red arrow indicates the right coronary artery, originating from the right coronary cusp. The blue arrow indicates the left main artery, originating from the left coronary cusp.

**Figure 4:**
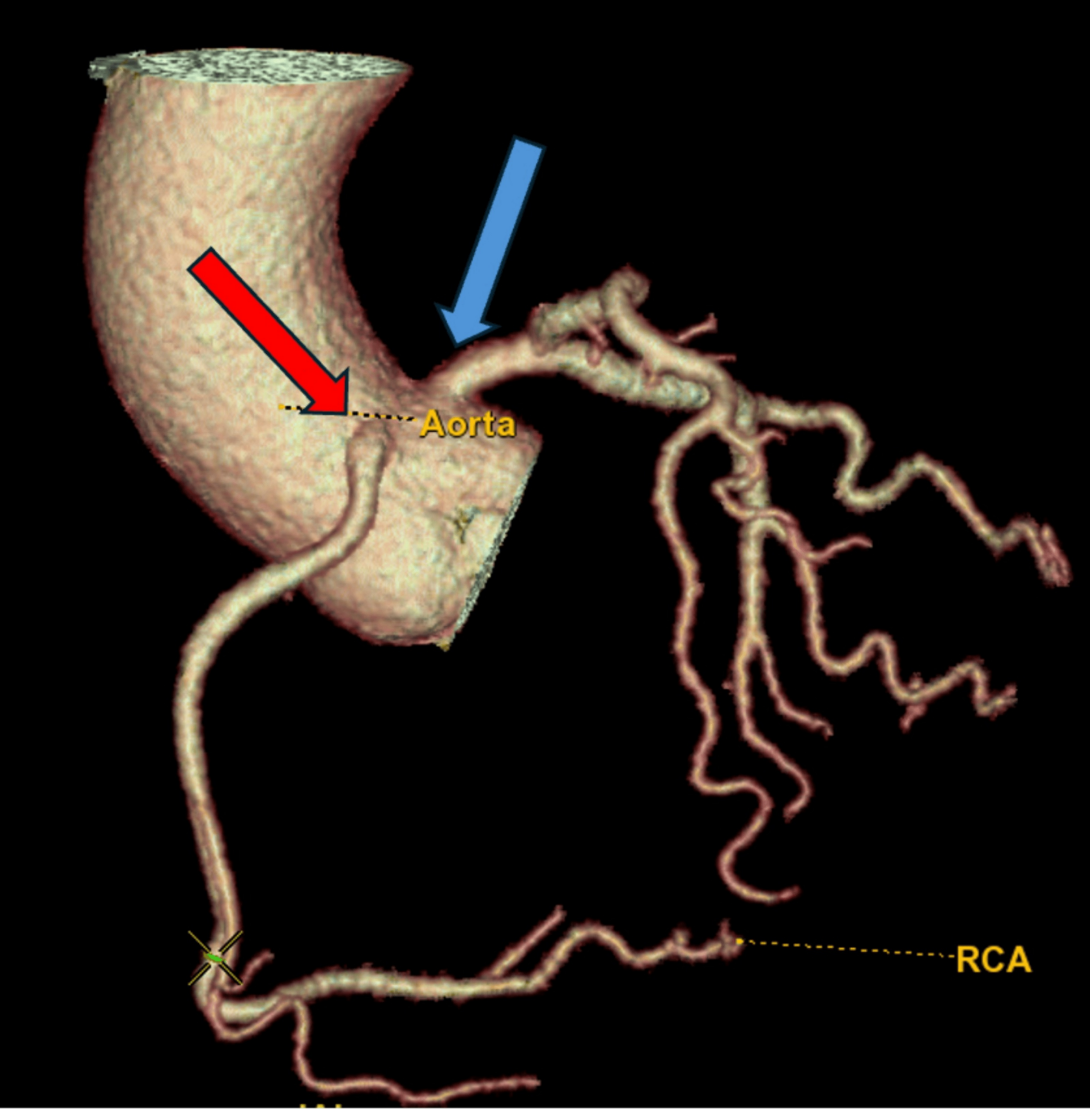
Coronary computed tomography angiography with a 3D-rendered image of the anomalous right coronary artery, originating from the left coronary cusp, anterior to the origin of the left main artery, and coursing anteriorly between the ascending aorta and the main pulmonary artery. The red arrow indicates the anomalous right coronary artery. The blue arrow indicates the left main artery.

The patient was first diagnosed with an anomalous RCA during cardiac catheterization 14 years prior to the current presentation. The catheterization revealed the RCA originating from the left coronary cusp, just anterior to the origin of the left main coronary artery, coursing anteriorly between the ascending aorta and the main pulmonary artery. It gave rise to a smaller right ventricular branch before tapering into a smaller vessel with a posterior descending artery branch. At that time, there were no signs of coronary artery disease in the RCA or the left main coronary artery and its branches. However, between the initial diagnosis and the current presentation, the patient experienced persistent episodes of chest pain, leading to multiple emergency department visits and hospitalizations. Prior to the current presentation, the patient underwent a recent stress echocardiogram, which was negative for ischemia and showed a normal ejection fraction of 60%-65%, along with mild aortic regurgitation. During her current hospitalization, the electrocardiogram revealed sinus bradycardia with a first-degree atrioventricular block. Further testing was performed after admission, including coronary computed tomography angiography (CTA), which showed the RCA arising as a high takeoff anteriorly and traversing normally without evidence of an inter-arterial course (Figures 1-4). No stenosis or disease was observed in any of the coronary arteries on the CTA. The patient was discharged on home medications, including metoprolol, aspirin, atorvastatin, and levothyroxine. She was also started on ranolazine 500 mg twice daily for microvascular disease and was educated on the importance of lifestyle modifications, including a sodium-restricted diet, weight management, and exercise as tolerated. She was advised to follow up regularly with her cardiologist. 

## Discussion

Multidetector computed tomography coronary angiography or coronary CTA is the gold standard for diagnosing an anomalous RCA [5]. However, transthoracic echocardiogram, magnetic resonance imaging, or coronary catheterization can also be used [1]. Once the diagnosis is confirmed, stress testing is performed to identify and quantify exertional ischemia, if present [1,5]. Exertion is an important factor to consider, as research has shown that the possible mechanisms for anginal symptoms, such as SCA, include incremental mechanical compression of the anomalous coronary artery by the great vessels, kinking caused by the sharp angulation of the anomalous artery, or an internal endothelial dysfunction causing vasospasm [4]. Furthermore, exertional ischemia is a critical factor when considering surgical intervention [4].

Patient-centered discussion and education are crucial in managing anomalous coronary arteries, given the range of treatment options. Surgery is indicated for symptomatic patients and asymptomatic patients with documented ischemia; however, these indications remain controversial [4]. Additionally, there are various surgical techniques, each dependent on the type of anomaly; therefore, no single technique is considered superior to others, as they are difficult to compare [1,3-5]. Furthermore, studies have shown that patients who undergo surgical interventions may experience recurrent chest pain postoperatively [4]. In asymptomatic patients and those without exertional ischemia, lifestyle modifications and medications may be considered. Lifestyle modifications include activity and exercise restrictions, a sodium-restricted diet, weight loss, and regular follow-up with a cardiologist. The use of medication therapy remains controversial, as only a few case reports and studies have described its use as a sole treatment option [7-9]. The most commonly used medications include beta-blockers, calcium channel blockers, nitrates, and anti-arrhythmic drugs [8,9]. This case highlights the controversial management of anomalous coronary arteries and underscores the need for further research on the comparison between medical and interventional/surgical management.

## Conclusions

Anomalies of the coronary arteries are rare congenital defects that can present with a variety of symptoms, from chest pain to SCA. However, some patients may be asymptomatic. Multidetector computed tomography coronary angiography or coronary CTA is considered the gold standard for diagnosing an anomalous RCA. Stress testing is commonly used to rule out exertional ischemia, which may necessitate surgical intervention if present. Management of anomalous coronary arteries remains controversial and depends on the patient's symptoms and specific anatomical variants. Our patient with an anomalous RCA had negative stress testing and, as a result, was managed with lifestyle modifications and medications, rather than surgical interventions. This case underscores the importance of patient-centered care and the need for further research to compare conservative management versus surgical intervention for anomalous coronary arteries.
